# Results From the United States Chronic Thromboembolic Pulmonary Hypertension Registry: Enrollment Characteristics and 1-Year Follow-up

**DOI:** 10.1016/j.chest.2021.05.052

**Published:** 2021-06-04

**Authors:** Kim M. Kerr, C. Greg Elliott, Kelly Chin, Raymond L. Benza, Richard N. Channick, R. Duane Davis, Feng He, Andrea LaCroix, Michael M. Madani, Vallerie V. McLaughlin, Myung Park, Ivan M. Robbins, Victor F. Tapson, Jeffrey R. Terry, Victor J. Test, Sonia Jain, William R. Auger

**Affiliations:** a University of California San Diego, La Jolla, CA; b University of Utah, Salt Lake City, UT and Intermountain Healthcare, Inc., Murray, UT; c University of Texas Southwestern, Dallas, TX; d The Ohio State University, Columbus, OH; e University of California Los Angeles, Los Angeles, CA; f AdventHealth, Orlando, FL; g University of Michigan, Ann Arbor, MI; h Virginia Mason Franciscan Health, Tacoma, WA; i Vanderbilt University Medical Center, Nashville, TN; j Cedars-Sinai Medical Center, Los Angeles, CA; k Texas Tech University Health Sciences, Lubbock, TX; l Temple University Hospital, Philadelphia, PA

**Keywords:** chronic thromboembolic pulmonary hypertension, CTEPH, pulmonary hypertension, registry, venous thromboembolism, 6MWD, 6-min walk distance, BPA, balloon pulmonary angioplasty, CTEPH, chronic thromboembolic pulmonary hypertension, DSA, digital subtraction angiography, mPAP, mean pulmonary artery pressure, PAH, pulmonary arterial hypertension, PE, pulmonary embolism, PH, pulmonary hypertension, PTE, pulmonary thromboendarterectomy, UCSD, University of California San Diego, US-CTEPH-R, United States Chronic Thromboembolic Pulmonary Hypertension Registry, WHO, World Health Organization

## Abstract

**Background:**

The United States Chronic Thromboembolic Pulmonary Hypertension Registry (US-CTEPH-R) was designed to characterize the demographic characteristics, evaluation, clinical course, and outcomes of surgical and nonsurgical therapies for patients with chronic thromboembolic pulmonary hypertension.

**Research Question:**

What are the differences in baseline characteristics and 1-year outcomes between operated and nonoperated subjects?

**Study Design and Methods:**

This study describes a multicenter, prospective, longitudinal, observational registry of patients newly diagnosed (< 6 months) with CTEPH. Inclusion criteria required a mean pulmonary artery pressure ≥ 25 mm Hg documented by right heart catheterization and radiologic confirmation of CTEPH. Between 2015 and 2018, a total of 750 patients were enrolled and followed up biannually until 2019.

**Results:**

Most patients with CTEPH (87.9%) reported a history of acute pulmonary embolism. CTEPH diagnosis delays were frequent (median, 10 months), and most patients reported World Health Organization functional class 3 status at enrollment with a median mean pulmonary artery pressure of 44 mm Hg. The registry cohort was subdivided into Operable patients undergoing pulmonary thromboendarterectomy (PTE) surgery (n = 566), Operable patients who did not undergo surgery (n = 88), and those who were Inoperable (n = 96). Inoperable patients were older than Operated patients; less likely to be obese; have a DVT history, non-type O blood group, or thrombophilia; and more likely to have COPD or a history of cancer. PTE resulted in a median pulmonary vascular resistance decline from 6.9 to 2.6 Wood units (*P* < .001) with a 3.9% in-hospital mortality. At 1-year follow-up, Operated patients were less likely treated with oxygen, diuretics, or pulmonary hypertension-targeted therapy compared with Inoperable patients. A larger percentage of Operated patients were World Health Organization functional class 1 or 2 at 1 year (82.9%) compared with the Inoperable (48.2%) and Operable/No Surgery (56%) groups (*P* < .001).

**Interpretation:**

Differences exist in the clinical characteristics between patients who exhibited operable CTEPH and those who were inoperable, with the most favorable 1-year outcomes in those who underwent PTE surgery.

**Clinical Trial Registration:**

ClinicalTrials.gov; No.: NCT02429284; URL: www.clinicaltrials.gov.

## Graphical Abstract

**Figure:**
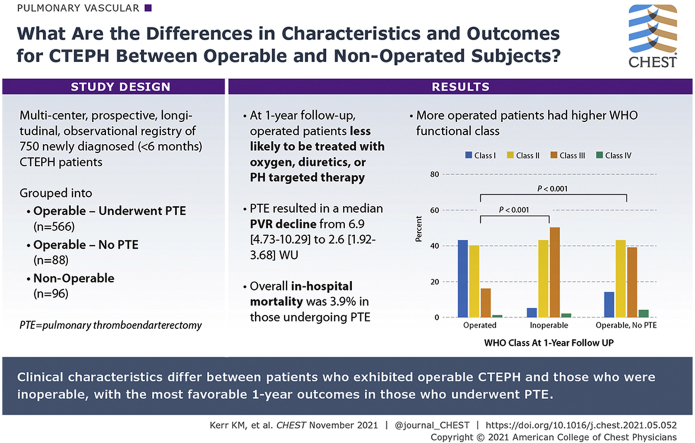

FOR EDITORIAL COMMENT, SEE PAGE 1599

Chronic thromboembolic pulmonary hypertension (CTEPH), an important diagnostic subgroup of pulmonary hypertension (PH), is characterized by the obstruction of pulmonary arteries with fibrotic, organized thrombus material and vascular remodeling resulting in PH and right ventricular failure.^1^ Although CTEPH shares some clinical and pathologic characteristics with pulmonary arterial hypertension (PAH), the etiology, diagnosis, and treatment of CTEPH differ substantially from those of PAH.

Although many registries have focused on PAH,^2^, ^3^, ^4^, ^5^, ^6^ there has not been a US multicenter registry focused on CTEPH. Investigators of the European CTEPH Registry described the epidemiology, risk factors, and outcomes of newly diagnosed European and Canadian patients with CTEPH.^7^^,^^8^ However, the evolution in medical and surgical approaches, including the availability of riociguat and balloon pulmonary angioplasty (BPA), as well as the differences in CTEPH management between the United States and Europe, provided the rationale to organize the first US CTEPH registry.

The United States Chronic Thromboembolic Pulmonary Hypertension Registry (US-CTEPH-R) is a contemporary CTEPH registry involving 30 PH centers. The current report describes the demographic characteristics, medical history, symptoms, timeline to diagnosis, risk factors, diagnostic approach, disease management, and 1-year outcomes of 750 US patients newly diagnosed with CTEPH.

## Patients and Methods

The US-CTEPH-R is a multicenter, prospective, longitudinal registry of patients newly diagnosed with CTEPH. The University of California San Diego (UCSD) is the sponsor and coordinating institution for the study, approved by the UCSD Human Research Protection Program (Project #141379). Thirty US sites (e-Appendix 1), selected based on a feasibility survey and geographic distribution, participated in the registry. Following provision of written informed consent, each patient was assigned a unique numerical patient-identifier to maintain confidentiality as required by the Health Insurance Portability and Accountability Act. The first patient was enrolled in April 2015, and target enrollment of 750 patients was met in March 2018. All subjects were followed up biannually until the last subject completed 1 year of follow-up in March 2019.

Consecutive patients diagnosed with CTEPH within 6 months of study consent and meeting the inclusion criteria were offered participation in the study. Time of diagnosis was defined as the date that the last of three hemodynamic and radiologic entry criteria for CTEPH (right heart catheterization, ventilation and perfusion lung scans, and pulmonary angiography) was met. Hemodynamic criteria included a mean pulmonary artery pressure (mPAP) ≥ 25 mm Hg measured by right heart catheterization and pulmonary capillary wedge pressure ≤ 15 mm Hg, or > 15 mm Hg if justified by the investigator. Radiologic criteria included mismatched perfusion defects on ventilation and perfusion scanning and findings compatible with chronic thromboembolic disease on digital subtraction (DSA), CT angiography, or magnetic resonance angiography. A physician adjudication team (e-Appendix 2) reviewed imaging, with two adjudicators agreeing independently that imaging met the predefined criteria for CTEPH. Subjects meeting enrollment criteria were followed up longitudinally. Patients who had previously undergone pulmonary thromboendarterectomy (PTE) or BPA were excluded.

All evaluations and procedures performed were at the discretion of the treating clinician. Data collected at enrollment are given in the text and tables. Longitudinal data were collected biannually during patient clinic visits or by patient telephone call and/or chart abstraction.

Descriptive analysis provided the median and first/third quartiles for continuous variables and a frequency table reported for categorical variables. For comparisons among the three groups, a Kruskal-Wallis test was performed for continuous variables and Fisher exact test for categorical variables. The Wilcoxon rank sum test and Fisher exact test were used for between-group (pairwise) comparisons when the overall group comparisons differed significantly (*P* < .05). To account for multiple testing, Bonferroni’s correction was applied, in which the critical *P* value for each pairwise between-group comparison was defined as *P* < .0167. Subgroup analysis of Operated and Operable/No Surgery patients comparing covariates of age, sex, race, and co-morbidities identified by the Fisher exact test to be significantly different between the groups was performed by using univariate logistic regression analysis. R 3.5.2 statistical software (www.rproject.org; R Foundation for Statistical Computing) was used for analysis.

## Results

**Figure 1:**
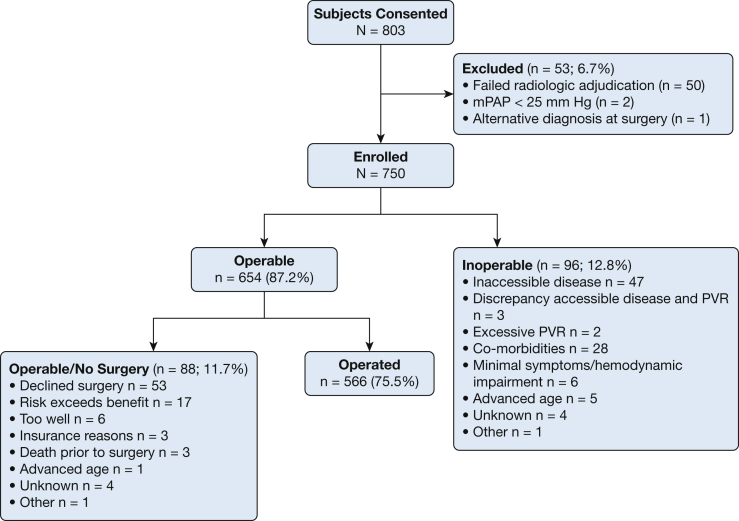
Disposition of subjects. Flow diagram for study participants. Subjects were assessed and deemed Operable or Inoperable. Operable subjects were further divided into those who underwent pulmonary thromboendarterectomy (Operated) and those who did not (Operable/No Surgery). mPAP = mean pulmonary artery pressure; PVR = pulmonary vascular resistance.

Between April 2015 and March 2018, a total of 803 patients consented to participate in the US-CTEPH-R. Fifty subjects (6.2%) failed radiologic adjudication. The most common reasons included: acute rather than chronic pulmonary embolism (PE; n = 18), no chronic PE (n = 16), or advanced parenchymal lung disease (n = 12). Three other subjects were excluded, resulting in 750 subjects in the final analysis (Fig 1) with subjects from all 50 US states (e-Appendix 3).

### Operability

All candidates were assessed for operability; 654 (87.2%) were deemed Operable and 96 (12.8%) deemed Inoperable. UCSD was the largest enroller (n = 218), but 71% of the subjects came from non-UCSD sites. Subjects enrolled at UCSD were more commonly Operated (91.8% vs 68.6%), compared with the 29 non-UCSD centers.

Inoperable patients included those with surgically inaccessible disease (49%), co-morbidities (29%), advanced age (5%), and minimal hemodynamic impairment (6.7%). Several patients presented multiple contraindications to surgery.

Of the 654 technically operable subjects, 88 (11.9%) did not undergo surgery. The most common explanations included subject refusing or delaying surgery (60%), risk exceeded benefit (19%) most commonly due to co-morbidities, and patient too well (6.8%) with multiple reasons for not undergoing surgery, including advanced age listed for some. The remaining 566 patients (74.3% of the total cohort) underwent PTE at 20 US centers, 18 of which were US-CTEPH-R sites.

Univariate logistic regression analysis identified advanced age (*P* < .001), non-Hispanic Black race (*P* = .043), the presence of COPD (*P* = .004), left ventricular diastolic (*P* = .012) or systolic (*P* = .021) dysfunction, a history of prolonged hospitalization (*P* = .021), and a history of leukemia/lymphoma (*P* = .023) or any cancer (*P* = .024) as covariates associated with technically operable patients not undergoing surgery (e-Table 1).

### Demographic Characteristics

**Figure 2:**
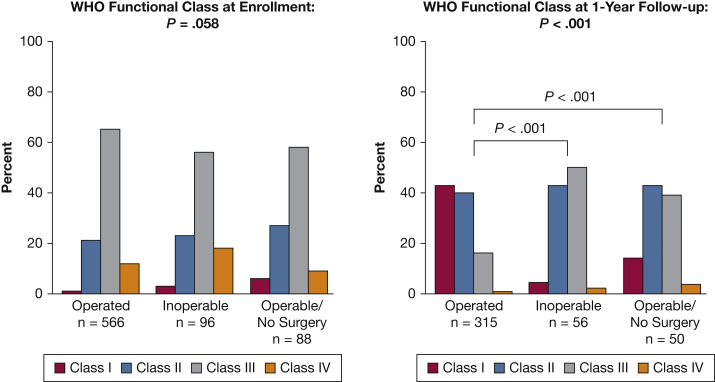
WHO functional class at enrollment and 1-year follow-up. WHO functional class is similar between the three cohorts at enrollment, but at 1-year follow-up Operated patients have a statistically better WHO functional class compared with both Inoperable and Operable/No Surgery patients (*P* < .001). WHO = World Health Organization.

Median age at enrollment was 59 years (range, 16-87 years). Male subjects comprised 50.8% of the total cohort (Table 1). Inoperable and Operable/No Surgery subjects were older than Operated subjects (both, *P* < .001). The median BMI was 29.7 kg/m^2^ (range, 12.3-58.7 kg/m^2^), and Inoperable subjects had a lower BMI. Most subjects were WHO functional class III at enrollment (Fig 2). There was no significant difference in WHO functional class or 6-min walk distance (6MWD) among the three groups.

**Table 1:** Baseline Demographic Characteristics

Characteristic	Total Cohort (N = 750)	Operated (n = 566)	Inoperable (n = 96)	Operable/No Surgery (n = 88)	*P* Value and Significance
Age, median [Q1-Q3], y	59 [46-69]	57 [44-67]	67 [56-74]	66 [52-73]	< .001^a^^,^^b^^,^^c^
Sex (% male)	50.8	52.7	40.6	50.0	.094
Race/ethnicity					.026^a^^,^^c^
Non-Hispanic white	492 (65.6%)	373 (65.9%)	65 (67.7%)	54 (61.4%)	
Non-Hispanic black	168 (22.4%)	120 (21.2%)	19 (19.8%)	29 (33%)	
Hispanic	44 (5.9%)	40 (7.1%)	4 (4.2%)	0 (0%)	
Other	46 (6.1%)	33 (5.8%)	8 (8.3%)	5 (5.7%)	
BMI, median [Q1-Q3], kg/m2	29.7 [25.8-35.9]	30.4 [26.1-36.3]	27.3 [23.8-34.1]	30.3 [26.4-35.3]	.006^a^^,^^b^
6MWD median [Q1-Q3], m	329 [235-400] n = 374	334 [235-406] n = 253	321 [244-376] n = 64	303 [228-389] n = 57	.390
WHO functional class I/II/III/IV, %	2/22/63/13	1/21/65/12	3/23/56/18	6/26/59/9	.058
Median time from symptom onset to suspicion of CTEPH, mo	10.1 [3.0-26.1]	12.1 [4.0-29.4]	7.0 [2.0-15.1]	6.0 [2.0-17.7]	< .001^a^^,^^b^^,^^c^

6MWD = 6-min walk distance; CTEPH = chronic thromboembolic pulmonary hypertension; PTE = pulmonary thromboendarterectomy; Q1-Q3 = first/third quartiles; WHO = World Health Organization.

a Comparison among three groups, *P* < .05 statistically significant.

b Between-group (Inoperable vs Operated) comparisons, *P* < .017 statistically significant.

c Between-group (Operable/No Surgery vs Operated) comparisons, *P* < .017 statistically significant.

### History of VTE Disease

The majority of patients with CTEPH (87.9%) reported a history of acute PE with (46.3%) or without (41.6%) DVT treated with anticoagulant agents. Few received systemic (2.3%) or catheter-directed (3.5%) thrombolytics. Almost one-half of the total CTEPH cohort reported recurrent episodes of PE (e-Table 2). The remaining patients either reported no history of acute PE or DVT (8.9%) or a history of DVT without PE (3.2%). Isolated upper extremity DVT was uncommon (1.6%). History of VTE in an immediate family member was reported in 17.7% of patients.

### Symptoms, Diagnostic Delays, and Co-morbidities

Reported cardiopulmonary symptoms included exertional dyspnea (91.1%) and/or dyspnea at rest (19.5%). Other commonly reported symptoms included edema (38%), chest discomfort (29%), dizziness (26%), fatigue (25.9%), cough (22.1%), palpitations (14.8%), and/or presyncope (10.7%). Patients experienced syncope (7.6%), anxiety (5.7%), and/or hemoptysis (4.5%) less frequently.

The median time from onset of cardiopulmonary symptoms until clinical suspicion of CTEPH was 10.0 months (interquartile range, 3.0-26.1 months) for the total study population. Although 70% of subjects reported persistent symptoms following treatment of their initial PE, the median time from PE to suspicion of CTEPH in this group with residual dyspnea was 13.1 months (interquartile range, 4.3-39 months). Cardiopulmonary symptoms were initially attributed to diagnoses other than CTEPH in 320 (43%) subjects. Alternative diagnoses included recurrent PE (n =131), asthma (n = 73), COPD (n = 60), PAH (n = 56), pneumonia (n = 45), and heart failure (n = 22).

Co-morbidities included obesity (49.2%), systemic hypertension (36.1%), sleep-disordered breathing (27.9%), previous major orthopedic surgery (18.1%), COPD (15.2%), diabetes (15.1%), asthma (12.3%), and coronary artery disease (10.9%) (e-Tables 3 and 4). Obesity was more common in both Operable cohorts. COPD co-existed more commonly with CTEPH in the Inoperable cohort (24%) and the Operable/No Surgery cohort (23.9%) than in the Operated cohort (12.4%). patients with CTEPH who underwent PTE were also less likely to report a history of cancer.

### Medical Therapy

**Figure 3:**
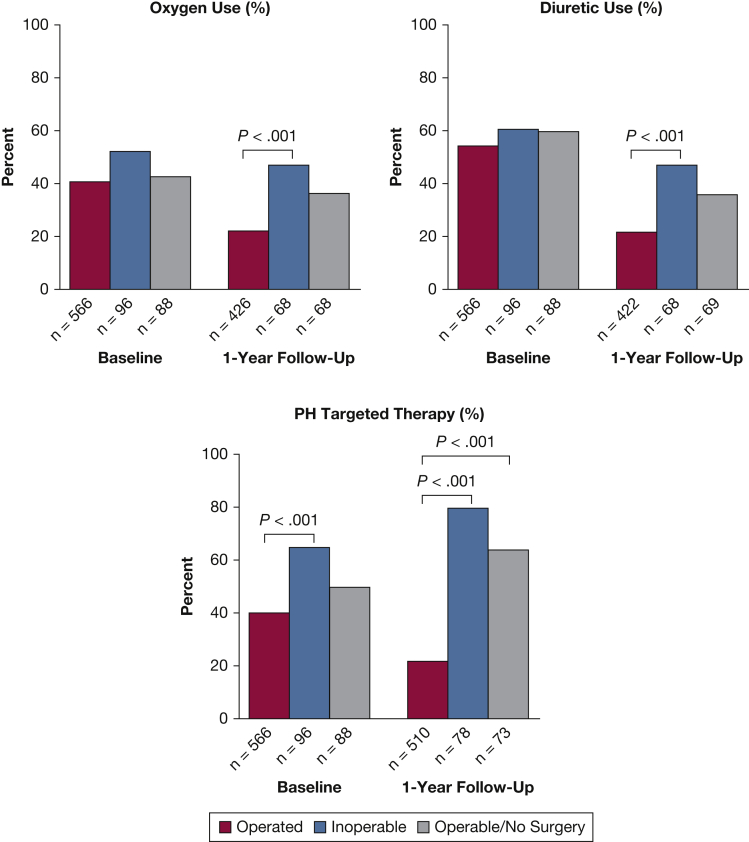
Medical therapy at enrollment and 1-year follow-up. At enrollment, there is no difference in the percentage of patients in each cohort using oxygen or diuretics. More Inoperable patients are on PH-targeted therapy than Operated patients at enrollment (*P* < .001). At 1-year follow-up, significantly more Inoperable patients are on diuretics and/or oxygen than Operated patients (*P* <.001) and more Inoperable and Operable/No Surgery patients are being treated with PH-targeted therapy compared with operated patients (*P* < .001). PH = pulmonary hypertension.

Medical therapy at enrollment (Fig 3) included diuretics (56%), supplemental oxygen (42%), and anticoagulants (98%). Warfarin (47%) was the most common anticoagulant, followed by direct oral anticoagulants (40%), low-molecular-weight heparins (9%), fondaparinux (1%), and aspirin alone (1%); no anticoagulant was reported in 2%.

At enrollment, 44% of subjects were receiving PH-targeted therapy; Inoperable patients were more likely than those deemed Operable to receive PH therapy (64.6% vs 39.4%; *P* < .001) (Table 2). Riociguat was prescribed most commonly (65%), followed by phosphodiesterase type 5 inhibitors (27%). Combination PH-targeted therapies were used to treat 15.5% of subjects.

**Table 2:** PH-Targeted Therapy at Enrollment

Therapy	Total (N = 750)	Operated (n = 566)	Inoperable (n = 96)	Operable/No Surgery (n = 88)	*P* Value and Significance
On any PH therapy	329 (43.9)	223 (39.4)	62 (64.6)	44 (50)	< .001^a^^,^^b^
Parenteral prostanoids	27 (8.2)	23 (10.3)	3 (4.8)	1 (2.3)	…
Inhaled prostanoids	2 (0.6)	2 (0.9)	0 (0)	0 (0)	…
ERA	31 (9.4)	21 (9.4)	5 (8.1)	5 (11.4)	…
PDE-5 inhibitor	89 (27.0)	61 (27.4)	16 (25.8)	12 (27.3)	…
Riociguat	215 (65.4)	141 (63.2)	41 (66.1)	33 (75)	…
Selexipag	1 (0.3)	0 (0)	1 (1.6)	0 (0)	…
Calcium channel blocker	16 (4.9)	12 (5.4)	1 (1.6)	3 (6.8)	…
Chronic inhaled NO	1 (0.3)	1 (0.4)	0 (0)	0 (0)	…
Oral treprostinil	2 (0.6)	1 (0.4)	1 (1.6)	0 (0)	…
> 1 PH-targeted therapy	51 (15.5)	36 (16.1)	5 (8.1)	10 (22.7)	…

Data on specific classes of drugs are presented as No. (%). n = total no. of subjects in cohort on pulmonary hypertension (PH)-targeted therapy (% of subjects in cohort on PH-targeted therapy). ERA = endothelin receptor antagonist; NO = nitric oxide; PDE-5 = phosphodiesterase type 5.

a Comparison among three groups, *P* < .05 statistically significant.

b Between-group (Inoperable vs Operated) comparisons, *P* < .017 statistically significant.

### CTEPH Diagnostic Evaluation

Ventilation-perfusion scintigraphy showed bilateral perfusion defects in 87.3% of the patients. Perfusion defects were larger than segmental in 51.4% of patients and segmental or smaller in 48.6%. More Inoperable and Operable/No Surgery subjects had small perfusion defects compared with Operated patients. Perfusion defects limited to one lung were seen more often in Operable/No Surgery subjects compared with the Operated and Inoperable patients (Table 3).

**Table 3:** Radiologic Imaging

Variable	Total (N = 750)	Operated (n = 566)	Inoperable (n = 96)	Operable/No Surgery (n = 88)	*P* Value and Significance
V/Q imaging					
Unilateral	12.7%	11.3%	11.5%	22.7%	.017^a^^,^^b^
Total occlusion	6.3%	6.9%	4.2%	4.6%	.564
Smaller defect	48.6%	43.0%	67.7%	63.6%	< .001^a^^,^^b^^,^^c^
CTA performed, no. (% of cohort)	388 (51.7)	262 (46.3)	66 (68.8)	60 (68.2)	< .001^a^^,^^b^^,^^c^
CTA distal disease	38.3%	37.2%	46.2%	35%	.358
DSA performed, no.(% of cohort)	436 (58.1)	365 (64.5)	40 (41.7)	31 (35.2)	< .001^a^^,^^b^^,^^c^
DSA distal disease	36.7%	29.3%	85%	61.3%	< .001^a^^,^^b^^,^^c^

CTA = CT angiography; distal disease = location of most proximal clot on angiography segmental or subsegmental; DSA = digital subtraction angiography; Smaller defect = largest perfusion defect segmental or subsegmental in size; Total occlusion= nonperfusion entire lung on ventilation and perfusion scan; Unilateral = perfusion defects confined to one lung.

a Comparison among three groups, *P* < .05 statistically significant.

b Between group (Operable/No Surgery vs Operated) comparisons, *P* < .017 statistically significant.

c Between group (Inoperable vs Operated) comparisons, *P* < .017 statistically significant.

According to CT angiography findings, the most proximal location of clot was in the main (19%), lobar (43%), segmental (30%), or subsegmental (8%) pulmonary arteries. DSA showed the most proximal clot in the main (12%), lobar (52%), segmental (31%), or subsegmental (5.7%) pulmonary artery levels. Distal disease, defined as the most proximal clot originating in one or more segmental or subsegmental pulmonary arteries, was noted more commonly in the Inoperable and Operable/No Surgery groups compared with the Operated group.

Cardiac catheterization revealed precapillary PH with a median mPAP of 44 mm Hg and a pulmonary vascular resistance of 6.86 Wood units (Table 4). Baseline hemodynamics were similar among the three cohorts, except that mPAP was higher in the Operated vs Operable/No Surgery group and cardiac index was lower in the Operated vs Inoperable groups.

**Table 4:** Enrollment Hemodynamics

Variable	Total Cohort (N = 750)	Operated (n = 566)	Inoperable (n = 96)	Operable/No Surgery (n = 88)	*P* Value and Significance
RAP, mm Hg	9 [6-13] (702)	9 [6-13] (533)	9 [5-14] (88)	9 [6-13] (81)	.801
mPAP, mm Hg	44 [36-51] (750)	44 [36-52] (566)	45 [34-50] (96)	40 [32-49] (88)	.024^a^^,^^b^
PCWP, mm Hg	12 [8-15] (731)	12 [8-15] (551)	11 [9-14] (93)	12 [9-14] (87)	.643
Cardiac output, L/min	4.59 [3.63-5.55] (737)	4.57 [3.6-5.57] (556)	4.68 [4.07-5.64] (94)	4.40 [3.6-5.42] (87)	.419
Cardiac index, L/min/m^2^	2.23 [1.84-2.7] (717)	2.2 [1.8-2.68] (542)	2.49 [2.1-2.88] (91)	2.2 [1.83-2.67] (84)	.003^a^^,^^c^
PVR, WU	6.86 [4.55-10.11] (722)	6.90 [4.73-10.29] (545)	6.33 [4.47-9.11] (91)	6.53 [3.67-10.09] (86)	.248

Data are presented as median [first-third quartiles] (number of subjects). Thermodilution values are used for reporting cardiac output and index whenever available; otherwise, Fick values are reported. mPAP = mean pulmonary artery pressure; PCWP = pulmonary capillary wedge pressure; PVR = pulmonary vascular resistance; RAP = right atrial pressure; WU = Wood units.

a Comparison among three groups, *P* < .05 statistically significant.

b Between group (Operable/No Surgery vs Operated) comparisons, *P* < .017 statistically significant.

c Between group (Inoperable vs Operated) comparisons, *P* < .017 statistically significant.

A thrombophilic disorder was identified in 37.9% of the 552 subjects tested, with antiphospholipid antibodies identified in 23%, activated protein C resistance/factor V Leiden (9.9%), and/or deficiencies of antithrombin III (4.9%), protein C (4.4%), or protein S (4.7%). Elevated factor VIII was found in 6.6% and elevated homocysteine levels in 5.8% of tested subjects. Blood group testing (n = 505) revealed that non-O type was more common in Operated (74.4%) and Operable/No Surgery (89.7%) patients compared with Inoperable patients (56.1%; *P* = .006). Six percent of the registry cohort had undergone splenectomy, 3.9% had a history of central venous catheter placement, and 1.6% had pacemaker placement (e-Table 4).

### Pulmonary Thromboendarterectomy

PTE was performed on 566 US-CTEPH-R patients. Proximal chronic thromboembolic disease (main, interlobar, or lobar pulmonary artery) was noted in most subjects, with involvement of the right lung (70%) more often than the left lung (54%). An additional surgical procedure was performed at the time of PTE in 184 subjects. Closure of a patent foramen ovale or atrial septal defect (n = 87) was the most common secondary procedure, followed by coronary artery bypass graft (n = 48) or tricuspid valve repair (n = 20). Early postoperative measurements showed significant improvement in all hemodynamic parameters compared with baseline (e-Table 5). Median mPAP decreased from 44 to 24 mm Hg, cardiac output increased from 4.57 to 5.5 L/min, and total pulmonary resistance decreased from 9.4 to 4.4 Wood units. Overall in-hospital mortality was 3.9% (n = 22), with causes of death attributed to right ventricular failure (n = 8), pulmonary hemorrhage (n = 7), reperfusion pulmonary edema (n = 4), other surgical hemorrhage (n = 3), recurrent thrombosis (n = 3), or other causes (n = 4).

Forty subjects (5.3%) underwent BPA, including 27 who were Inoperable, 6 who were operable but did not undergo PTE, and 7 who underwent both PTE and BPA.

### One-Year Follow-up

Early discontinuation at 1 year occurred in 56 Operated subjects (death, n = 30; lost to follow-up, n = 24; withdrew, n = 1; other, n = 1), 18 Inoperable patients (death, n = 11; lost to follow-up, n = 6; other, n = 1), and 15 Operable/No Surgery patients (death, n = 8; lost to follow-up, n = 6; other, n = 1).

Although WHO functional class distribution was similar in the three groups at baseline, at 1-year follow-up, the Operated group had a statistically better WHO functional class compared with the Inoperable and Operated/No Surgery cohorts (*P* < .001) (Fig 2). More of the Operated group were WHO functional class I or II at 1 year (82.9%) compared with the Inoperable (48.2%) or Operable/No Surgery (56%) cohorts (*P* < .001). However, all three cohorts showed functional class improvements at 1 year.

Fewer Operated subjects were on oxygen, diuretics, or PH-targeted therapy compared with the Inoperable group at 1 year (*P* < .001). The use of PH-targeted therapy decreased from baseline in the Operated group and increased at 1 year in the Inoperable and Operated/No Surgery groups (Fig 3).

One-year mortality differed for the three cohorts (Operated, 5.6%; Operable/No Surgery, 9.9%; and Inoperable, 12.4%; *P* = .028). Most deaths in the Operated group were perioperative, with three deaths following discharge attributed to thromboembolism. Six of the 10 deaths in the Inoperable group were attributed to worsening PH/right heart failure, and five of the eight deaths in the Operable/No Surgery group were attributed to worsening PH/right heart failure with one death due to hemorrhage.

## Discussion

The US-CTEPH-R is the first prospective, multicenter, observational registry of US patients newly diagnosed with CTEPH. The US-CTEPH-R provides a contemporary profile of US patients with CTEPH, including epidemiologic and clinical features, results of diagnostic tests, medical therapy, characteristics of patients selected for PTE and those deemed Inoperable, and outcomes 1 year after enrollment.

An initial observation from the registry was that the diagnosis of CTEPH can be difficult. Despite study sites being experienced PH centers, 50 patients initially diagnosed with CTEPH (6.2%) failed adjudication, the differentiation of acute PE from chronic thromboembolic disease the most common reason for adjudication failure.

As has been reported previously,^7^^,^^10^ diagnostic delays were common, with a median period of 10 months following symptom onset, and even a longer period of 13.1 months following an acute PE episode, prior to CTEPH consideration. Clinicians initially attributed the cardiopulmonary symptoms of almost one-half of patients to co-morbid diagnoses, such as obesity, COPD, or asthma. The potential consequence of doing so was that patients were often diagnosed at an advanced stage of disease. At enrollment, most subjects were WHO functional class III, and > 75% of subjects had a mPAP > 35 mm Hg with elevated pulmonary vascular resistance and reduced cardiac index. More than one-half of the patients required diuretics, and almost one-half were treated with PH-targeted therapy and/or supplemental oxygen.

Most registry patients reported a history of acute PE, with only 8.9% reporting no prior VTE. Patients commonly described incomplete resolution of symptoms following an acute PE, and almost one-half of the subjects reported a history of recurrent PE while taking an anticoagulant. These observations imply that a component of chronic PE may have been present at the time of an “acute PE,” as has been reported by others.^9^

Co-morbidities associated with CTEPH described in other cohort publications^9^^,^^11^^,^^12^ were similarly observed in our registry, including a history of thrombophilia, cancer, non-type O blood group, and splenectomy.

In this registry, 87.2% of patients with CTEPH were deemed operable. Inoperable patients were older than Operated patients, and less likely to be obese, have a history of DVT, non-type O blood group, or thrombophilia, and more likely to have COPD and/or a history of cancer. As with the Inoperable patients, Operable/No Surgery patients were older and more likely to have COPD, left ventricular dysfunction, and/or a history of cancer than the Operated patients with CTEPH. WHO functional class, 6MWD, and hemodynamics were not different between the Operable/No Surgery and Operated groups. However, as with the Inoperable subjects, diagnostic delay was shorter in the Operable/No Surgery group compared with the Operated group, conceivably related to more touchpoints with the health care system due to advanced age and co-morbidities. Operable/No Surgery patients also had smaller perfusion defects and distal disease on DSA than the Operated group, raising the possibility that less impressive disease burden and uncertainty of surgical benefit influenced the decision not to proceed with surgery.

PH-targeted therapy was more common in Inoperable subjects compared with Operated subjects. Although there are no data to support bridging therapy, almost 40% of the Operated subjects received PH-targeted therapy prior to surgery.

In the aggregate, registry data suggest that PTE benefits most patients with operable CTEPH. At the 1-year follow-up, an improvement was seen in WHO functional class in all three groups. However, the Operated group experienced greater improvement in functional class, decreased use of medical therapy, and improved survival compared with the other two groups.

Compared with the median age in the European CTEPH Registry,^7^ the median age of the US-CTEPH-R subjects was slightly younger (59 vs 63 years), but 6MWD and functional class distributions were similar. A significant difference is seen in the racial/ethnic diversity of the populations, with the European CTEPH Registry reporting 95.9% White subjects and the US-CTEPH-R 65.6% non-Hispanic White and 22.4% non-Hispanic Black subjects. The US registry revealed a higher prevalence of obesity (49% vs 9%), sleep-disordered breathing (27.9% vs 3.1%), and diabetes (15.1% vs 5.2%). Patients in the US-CTEPH-R more often reported a history of acute PE (87.9% vs 74.8%) and recurrent PE (48.5% vs 32.8%), yet a lower incidence of DVT (49.5% vs 56.1%). Both registries observed that Inoperable subjects were less likely to have a history of DVT compared with Operable subjects. US-CTEPH-R patients were more likely to report a family history of VTE (17.7% vs 6.6%), antiphospholipid antibodies (23% vs 10.1%), and splenectomy (6.1% vs 3.4%).

Enrollment hemodynamics and the percentage of all subjects with distal disease (37%-40%) on angiographic imaging were similar between the US-CTEPH-R and the European CTEPH Registry. In this registry, 87.2% of the subjects were deemed operable (75.5% underwent PTE) in contrast to the 63.3% reported operable (56.8% underwent PTE) in the European CTEPH Registry. Possible reasons for the difference include the pre-enrollment adjudication of subjects in the US-CTEPH-R, differences in the definition of operability, and a large number of surgical centers participating in the US-CTEPH-R. For example, at UCSD, only 5.5% of the enrolled subjects were deemed Inoperable, reflecting a referral bias to a large surgical center.

Several study limitations have been identified. This analysis was an attempt at a “real-world” description of the evaluation of patients with CTEPH in the United States. There are differences between centers in what defines technical operability as well as co-morbidities that prohibit surgical candidacy. This resulted in some overlap between the Inoperable and Operable/No Surgery groups. US-CTEPH-R sites are academic PH centers, offering more advanced PAH and CTEPH therapies. Hence, the population enrolled by this registry likely does not reflect the CTEPH population cared for in the community without referral to a PH center. Many of the sites were also surgical centers, which may increase the percentage of Operable patients in the US-CTEPH-R. BPA was a newly emerging therapy in the United States during the conduct of the trial, and the small number of BPA subjects in the US-CTEPH- R does not likely reflect the role BPA currently plays in the management of CTEPH. Medical history was collected through chart abstraction and patient interview, with potential for recall errors. In addition, because US-CTEPH-R was observational, not all data elements were available for 1-year follow-up. One-year follow-up data are presented as descriptive statistics. Longer term follow-up data will be presented in a future publication and will include adjustments for covariates that may affect outcomes.

## Interpretation

The US-CTEPH-R provides a contemporary description of US patients with CTEPH, their evaluation, management, and 1-year outcomes. Important observations include difficulties in making a timely diagnosis of CTEPH and the presence of co-morbidities that may mask the diagnosis and alter management. Nevertheless, US-CTEPH-R data show that PTE produces favorable outcomes with a low perioperative mortality for selected patients with CTEPH.

**Take-home Points**

**Study Question:** What are the demographic characteristics, risk factors, diagnostic findings, treatment patterns, and 1-year outcomes of patients with CTEPH, and are there differences between operated and nonoperated patients?

**Results:** US patients with CTEPH are predominantly middle-aged men and women with a history of PE. The diagnosis of CTEPH was often delayed because CTEPH symptoms were attributed to co-morbid conditions. Most patients in the US-CTEPH-R underwent PTE, with a 3.9% in-hospital mortality rate. PTE patients were younger, more likely of non-Hispanic White race, and less likely to have co-morbidities than technically operable patients who did not undergo PTE. One-year outcomes were more favorable in Operated patients.

**Interpretation:** The US-CTEPH-R provides a contemporary description of US patients with CTEPH, their evaluation, management, and 1-year outcomes. The registry also offers the opportunity for future investigation into the impact of co-morbidities and medical therapies on long-term outcomes.

## Supplementary Data

e-Online Data
