# Supplementary material for: Results From the United States Chronic Thromboembolic Pulmonary Hypertension Registry: Enrollment Characteristics and 1-Year Follow-up
Source: Chest. 2021 Jun 4;160(5):1822–31. doi: 10.1016/j.chest.2021.05.052 (PMC8628169; doi:10.1016/j.chest.2021.05.052)
Supplement: e-Online Data [file mmc1.pdf]

CHEST Online Supplement

Results From the United States Chronic Thromboembolic Pulmonary Hypertension Registry

Enrollment Characteristics and 1-Year Follow-up

Kim M. Kerr, MD, FCCP; C. Greg Elliott, MD; Kelly Chin, MD; Raymond L. Benza, MD; Richard N. Channick, MD; R. Duane Davis, MD; Feng He, PhD; Andrea LaCroix, PhD; Michael M. Madani, MD; Vallerie V. McLaughlin, MD; Myung Park, MD; Ivan M. Robbins, MD; Victor F. Tapson, MD; Jeffrey R. Terry, MBA; Victor J. Test, MD; Sonia Jain, PhD; and William R. Auger, MD

CHEST 2021; 160(5):1822-1831

Online supplements are not copyedited prior to posting and the author(s) take full responsibility for the accuracy of all data.

© 2021 AMERICAN COLLEGE OF CHEST PHYSICIANS. Reproduction of this article is prohibited without written permission from the American College of Chest Physicians. See online for more details. **DOI:** 10.1016/j.chest.2021.05.052

e-Table 1: Baseline Characteristics Associated with Technically Operable Patients Not Undergoing Surgery

	OR	95% CI	*P* Value and Significance
Older age	0.96	0.95-0.98	<0.0001[a]
Gender	1.09	0.69-1.71	0.706
Non-Hispanic black race[b]	1.67	1.02-2.74	0.043 [a]
Chronic obstructive pulmonary disease	0.45	0.26-0.78	0.004 [a]
Left ventricular diastolic dysfunction	0.27	0.10-0.75	0.012 [a]
Left ventricular systolic dysfunction	0.15	0.03-0.76	0.022 [a]
Prolonged hospitalization[c]	0.50	0.28-0.90	0.021 [a]
History of leukemia or lymphoma	0.23	0.06-0.81	0.023 [a]
History of solid organ cancer	0.53	0.25-1.11	0.092
History of any cancer[d]	0.46	0.24-0.90	0.024 [a]
Obesity, BMI > 30	1.00	0.64-1.56	0.989
Extreme obesity, BMI > 40	1.09	0.58-2.05	0.787
PH-targeted therapy at enrollment	0.65	0.41-1.02	0.061

OR = odds ratio, CI = confidence interval

[a]Univariate logistic regression analysis comparing Operable/No surgery patients to Operated patients. $P < 0.05$ statistically significant

[b]Non-Hispanic black race compared to non-Hispanic white race

[c]Prolonged hospitalization (> 2 weeks within 5 years of symptom onset)

[d]Excluding non-melanoma skin cancer

Online supplements are not copyedited prior to posting and the author(s) take full responsibility for the accuracy of all data.

e-Table 2: Acute Thromboembolic History

	Total cohort N=750	Operated n=566	Inoperable n=96	Operable/no surgery n=88	*P* Value and Significance
Acute PE n (%)	659 (87.9%)	505 (89.2%)	80 (83.3%)	74 (84.1%)	0.125
Acute DVT	371 (49.5%)	297 (52.5%)	37 (38.5%)	37 (42.1%)	0.014[ab]
Acute PE + DVT	347 (46.3%)	280 (49.5%)	32 (33.3%)	35 (39.8%)	0.006[ab]
Acute PE, no DVT	312 (41.6%)	225 (39.8%)	48 (50.0%)	39 (44.3%)	0.151
Acute DVT, no PE	24 (3.2%)	17 (3.0%)	5 (5.2%)	2 (2.3%)	0.437
No acute DVT or PE	67 (8.9%)	44 (7.8%)	11 (11.5%)	12 (13.6%)	0.114
Upper extremity DVT	12 (1.6%)	9 (1.6%)	2 (2.1%)	1 (1.1%)	0.880
Recurrent PE	319 (48.5%)	249 (49.4%)	33 (41.3%)	37 (50.0%)	0.386

PE = pulmonary embolism, DVT = deep venous thrombosis
Percentage of recurrent PE Based on number in cohort with history of acute PE
[a]Comparison among three groups, $P < 0.05$ statistically significant
[b]Between group (Inoperable vs Operated) comparisons, $P < 0.017$ statistically significant

Online supplements are not copyedited prior to posting and the author(s) take full responsibility for the accuracy of all data.

e-Table 3: Co-morbidities

	Total cohort N=750	Operated n=566	Inoperable n=96	Operable/no surgery n=88	*P* Value and Significance
Obesity BMI > 30	369 (49.2%)	289 (51.1%)	35 (36.5%)	45 (51.1%)	0.027[ab]
Extreme obesity BMI >40	108 (14.4%)	90 (15.9%)	5 (5.2%)	13 (14.8%)	0.013[ab]
COPD	114 (15.2%)	70 (12.4%)	23 (24.0%)	21 (23.9%)	<0.001[abc]
LV systolic dysfunction	7 (0.9%)	3 (0.5%)	1 (1.0%)	3 (3.4%)	0.030[a]
LV diastolic dysfunction	20 (2.7%)	11 (1.9%)	3 (3.1%)	6 (6.8%)	0.032[a]
Congenital heart dis	22 (2.9%)	14 (2.5%)	3 (3.1%)	5 (5.7%)	0.238
Valvular heart disease	20 (2.7%)	13 (2.3%)	2 (2.1%)	5 (5.7%)	0.197
Systemic hypertension	271 (36.1%)	202 (35.7%)	35 (36.5%)	34 (38.6%)	0.857
Coronary artery disease	82 (10.9%)	54 (9.5%)	13 (13.5%)	15 (17.1%)	0.069
Atrial fibrillation	53 (7.1%)	40 (7.1%)	5 (5.2%)	8 (9.1%)	0.581
History of stroke/TIA	41 (5.5%)	29 (5.1%)	7 (7.3%)	5 (5.7%)	0.585
Interstitial lung disease	12 (1.6%)	8 (1.4%)	2 (2.1%)	2 (2.3%)	0.685
Sleep disorder breathing	209 (27.9%)	164 (29.0%)	21 (21.9%)	24 (27.3%)	0.362
Asthma	92 (12.3%)	76 (13.4%)	8 (8.3%)	8 (9.1%)	0.269
Diabetes	113 (15.1%)	77 (13.6%)	17 (17.7%)	19 (21.6%)	0.100
Scleroderma	2 (0.3%)	1 (0.2%)	1 (1.0%)	0 (0.0%)	0.431
Systemic lupus erythematosus	17 (2.3%)	15 (2.7%)	1 (1.0%)	1 (1.1%)	0.644
Portal hypertension	6 (0.8%)	4 (0.7%)	1 (1.0%)	1 (1.1%)	0.455
Inflammatory bowel dis	11 (1.5%)	7 (1.2%)	3 (3.1%)	1 (1.1%)	0.233
Renal disease	10 (1.3%)	6 (1.1%)	1 (1.0%)	3 (3.4%)	0.147
Toxins/drugs	36 (4.8%)	28 (5.0%)	4 (4.2%)	4 (4.6%)	>0.999

Renal disease = End stage renal disease on dialysis or nephrotic syndrome
Toxins/drugs = amphetamines, amphetamine like drugs, cocaine, fenfluramine, or dexfenfluramine
[a]Comparison among three groups, $P < 0.05$ statistically significant
[b]Between group (Inoperable vs Operated) comparisons, $P < 0.017$ statistically significant
[c]Between group (Operable/No surgery vs Operated) comparisons, $P < 0.017$ statistically significant

Online supplements are not copyedited prior to posting and the author(s) take full responsibility for the accuracy of all data.

e-Table 4: Co-morbidities Associated with CTEPH and Venous Thromboembolism

	Total cohort N=750	Operated n=566	Inoperable n=96	Operable/no surgery n=88	*P* Value and Significance
H/o VTE in family member	17.7%	19.3%	13.6%	11.7%	0.169
Thrombophilia (any)<br>N tested	209 (37.9%)<br>n=552	175 (39.2%)<br>n=446	19 (33.3%)<br>n=57	15 (30.6%)<br>n=49	0.400
LAC/APA<br>N tested	120 (23.0%)<br>n=522	103 (24.3%)<br>n=424	9 (17.3%)<br>n=52	8 (17.4%)<br>n=46	0.378
Non Type O Blood Group±<br>N tested	372 (73.8%)<br>n=504	323 (74.4%)<br>n=434	23 (56.1%)<br>n=41	26 (89.7%)<br>n=29	0.006[abc]
Venous insufficiency	19 (2.5%)	15 (2.7%)	4 (4.2%)	0 (0.0%)	0.178
Post thrombotic syndrome	53 (7.1%)	39 (6.9%)	9 (9.4%)	5 (5.7%)	0.563
H/o solid organ cancer	58 (7.7%)	36 (6.4%)	12 (12.5%)	10 (11.4%)	0.042[a]
H/o any cancer	69 (9.2%)	42 (7.4%)	14 (14.6%)	13 (14.8%)	0.013[a]
H/o leukemia/lymphoma	12 (1.6%)	6 (1.1%)	2 (2.1%)	4 (4.6%)	0.034[a]
H/o myeloproliferative disease	19 (2.5%)	11 (1.9%)	6 (6.3%)	2 (2.3%)	0.055
Splenectomy	46 (6.1%)	29 (5.1%)	10 (10.4%)	7 (8.0%)	0.094
Prolonged hospitalization	99 (13.2%)	65 (11.5%)	16 (16.7%)	18 (20.5%)	0.040[a]
Major orthopedic surgery	136 (18.1%)	96 (17.0%)	23 (24.0%)	17 (19.3%)	0.231
Central venous catheter placement	29 (3.9%)	18 (3.2%)	7 (7.3%)	4 (4.6%)	0.143
Chronic pacemaker implantation	12 (1.6%)	10 (1.8%)	1 (1.0%)	1 (1.1%)	>0.999
Ventriculoatrial shunt	2 (0.3%)	2 (0.4%)	0 (0.0%)	0 (0.0%)	>0.999
Hypothyroid on supplements	83 (11.1%)	63 (11.1%)	7 (7.3%)	13 (14.8%)	0.046[a]
Chronic infectious disease	4 (0.5%)	3 (0.5%)	0 (0%)	1 (1.1%)	0.456
Tobacco use (any)	302 (40.3%)	219 (38.7%)	45 (46.9%)	38 (43.2%)	0.264

LAC/APA= lupus anticoagulant, antiphospholipid antibodies
Percentage thrombophilia and LAC base upon number of subjects tested in each cohort
Any cancer = Any solid organ cancer, melanoma, lymphoma or leukemia
Chronic infectious disease = HIV or osteomyelitis
[a]Comparison among three groups, $P < 0.05$ statistically significant
[b]Between group (Inoperable vs Operable/No surgery) comparisons, $P < 0.017$ statistically significant
[c]Between group (Inoperable vs Operated) comparisons, $P < 0.017$ statistically significant

Online supplements are not copyedited prior to posting and the author(s) take full responsibility for the accuracy of all data.

e-Table 5: Pre and Early Post-operative Hemodynamics

	Baseline	Post Operative	*P* Value and Significance
RAP (mmHg)	9 [6-13] (533)	9 [6-12] (500)	0.0028[a]
mPAP (mmHg)	44 [36-52] (566)	24 [20-30] (554)	< 0.001[a]
PCWP (mmHg)	12 [8-15] (551)	Not measured	
Cardiac output (L/min)	4.57 [3.6-5.6] (556)	5.5 [4.6-6.6] (520)	<0.001[a]
Cardiac index(L/min/m^2)	2.2 [1.8-2.7] (542)	2.7 [2.4-3.1] (510)	< 0.001[a]
PVR (WU)	6.9 [4.73-10.29] (545)	2.6 [1.92-3.68] (465)	< 0.001[a]
TPR (WU)	9.44 [7.12-13.32] (556)	4.4 [3.33-5.83] (515)	< 0.001[a]

All values expressed as median [first - third quartiles] (number of subjects)
RAP = right atrial pressure, mPAP = mean pulmonary artery pressure, PCWP = pulmonary capillary wedge pressure, PVR = pulmonary vascular resistance, TPR = total pulmonary resistance
Post-operative PVR calculated substituting the RAP for the PCWP
Thermodilution values used for reporting cardiac output and index whenever available, otherwise Fick value reported
Wilcoxon Signed-Rank Test is performed to compare between baseline and post-operative
[a]$P < 0.05$ statistically significant

Online supplements are not copyedited prior to posting and the author(s) take full responsibility for the accuracy of all data.

e-Appendix 1: US-CTEPH-R Study Sites and Principal Investigators

Site ID	Institution Name	Principal Investigator
1	University of California San Diego	Kim Kerr, MD
2	Cedars-Sinai, Los Angeles	Victor Tapson, MD
3	Cleveland Clinic	Gustavo Heresi, MD
4	Massachusetts General Hospital	Josanna Rodriguez-Lopez, MD
5	Columbia University Medical Center	Erika Berman-Rosenzweig, MD
6	Louisiana State University	Matthew Lammi, MD
7	University of Chicago	Remzi Bag, MD
8	University of Maryland	Gautam Ramani, MD
9	University of Pennsylvania	Harold Palevsky, MD
10	University of Kansas	Timothy Williamson, MD
11	Stanford University	Roham Zamanian, MD
12	University of Colorado, Denver	Todd Bull, MD
13	Mayo Clinic, Jacksonville	Charles Burger, MD
14	Emory University	Micah Fisher, MD
15	Mayo Clinic, Rochester	Michael Krowka, MD
16	Allegheny Health	Manreet Kanwar, MD
17	University of Michigan	Vallerie McLaughlin, MD
18	Virginia Commonwealth University	Daniel Grinnan, MD
19	Washington University, St. Louis	Murali Chakinala, MD
20	University of Washington, Seattle	Peter Leary, MD, PhD
21	University of Texas, Southwestern	Kelly Chin, MD
22	University of Florida	Hassan Alnuaimat, MD
23	Brigham & Women's Hospital	Aaron Waxman, MD
24	Duke University Medical Center	Sudarshan Rajagopal, MD, PhD
25	Vanderbilt University	Ivan Robbins, MD
26	Aurora Health Care	Dianne Zwicke, MD
27	University of California Los Angeles	Rajan Saggar, MD
28	Intermountain Medical Center	Lynnette Brown, MD
29	Temple University	Paul Forfia, MD
30	Houston Methodist Hospital	Zeenat Safdar, MD

e-Appendix 2: Members of the Radiologic Adjudication Committee

William R. Auger, MD, Temple University
Richard N. Channick, MD, University of California Los Angeles
Kim M. Kerr, MD, University of California San Diego
Michael M. Madani, MD, University of California San Diego
Ivan M. Robbins, MD, Vanderbilt University
Victor J. Test, MD, Texas Tech University

Online supplements are not copyedited prior to posting and the author(s) take full responsibility for the accuracy of all data.

e-Appendix 3: Subject State of Residence (using first 3 digits of subject ZIP code)

**Subject State of Residence**	**Number Enrolled (% subjects), N = 746**
Alabama	1 (0.1)
Alaska	1 (0.1)
Arizona	8 (1.1)
Arkansas	8 (1.1)
California	77 (10.3)
Colorado	31 (4.2)
Connecticut	9 (1.2)
Delaware	1 (0.1)
District of Columbia	1 (0.1)
Florida	33 (4.4)
Georgia	25 (3.4)
Hawaii	1 (0.1)
Idaho	10 (1.3)
Idaho + Wyoming	3 (0.4)
Illinois	19 (2.5)
Indiana	18 (2.4)
Iowa	7 (0.9)
Kansas	7 (0.9)
Kentucky	10 (1.3)
Louisiana	8 (1.1)
Maine	9 (1.2)
Maryland	23 (3.1)
Massachusetts	23 (3.1)
Michigan	36 (4.8)
Minnesota	9 (1.2)
Mississippi	4 (0.5)
Missouri	22 (2.9)
Montana	1 (0.1)
Nebraska	3 (0.4)
Nevada	8 (0.7)
New Hampshire	5 (1.1)
New Jersey	17 (2.3)
New Mexico	11 (1.5)
New York	40 (5.4)
North Carolina	30 (4.0)
North Dakota	3 (0.4)
Ohio	29 (3.9)
Oklahoma	5 (0.7)
Oregon	6 (0.8)
Pennsylvania	33 (4.4)
Rhode Island	4 (0.5)
South Carolina	9 (1.2)
South Dakota	4 (0.5)
Tennessee	14 (1.9)
Texas	48 (6.4)
Utah	23 (3.1)
Vermont	6 (0.8)
Virginia	17 (2.3)
Washington	11 (1.5)
West Virginia	4 (0.5)
Wisconsin	10 (1.3)
Wyoming	1 (0.1)
Unknown	4

Online supplements are not copyedited prior to posting and the author(s) take full responsibility for the accuracy of all data.
